# Exploring the pros and cons of new approaches for gamete cross-border donation based on fresh and vitrified oocytes

**Published:** 2020-08-05

**Authors:** A La Marca, M Capuzzo, S Bartolucci, F Schirinzi, MB Dal Canto, J Buratini, M Mignini Renzini, A Rodriguez, R Vassena

**Affiliations:** Clinica EUGIN, Via Nobili 188/F, 41126 Modena, Italy;; Department of Medical and Surgical Sciences for Mother, Child and Adult, University of Modena and Reggio Emilia, Largo del Pozzo 71, 41123 Modena, Italy;; Biogenesi Reproductive Medicine Centre, Istituti Clinici Zucchi, Via Zucchi 24, 20052 Monza, Italy;; Department of Physiology, Institute of Biosciences, Sao Paulo State University, Rua Prof. Antonio Celso Wagner Zanin 250, 18618689, Botucatu, Brazil;; Clínica EUGIN, Carrer de Balmes 236, 08006 Barcelona, Spain.

**Keywords:** IVF, oocyte donation, vitrified oocytes, frozen embryos, crossborder

## Abstract

As highlighted by European statistics, the employment of donor oocytes is a growing option for women who cannot make use of their own gametes. As the potential recipients are continuously increasing in number, a donor programme which satisfies this demand is mandatory. Improvements in cryopreservation techniques, like oocyte and embryo vitrification, have led to the overcoming of the sequence of stimulation-retrieval-transfer both from a spatial and a temporal point of view, with the development of cryobanks of oocytes permitting crossborder donation. However, while some studies report comparable success when using vitrified and fresh oocytes we still need to investigate whether the use of fresh oocytes give higher live birth rate than cryopreserved ones, when the same number of oocytes are given. The performance of embryo cryopreservation, conversely, seems to be more reliable. A novel approach based on the shipment of frozen sperm from the recipient’s country to the oocyte donor’s one, where fresh oocytes are inseminated and the resulting embryos frozen and transported back to the referring IVF centre to perform a frozen embryo transfer may be a good strategy. We believe that the use of frozen embryos from fresh oocytes could be associated with a higher cumulative live birth rate per cycle, while favouring personalised oocyte recipient care with a flexible number of oocytes assigned and limiting the burden of travelling abroad.

## Introduction: an overview of oocyte donation

In vitro fertilization (IVF) with donor oocytes is an increasingly widespread therapeutic option for women who cannot make use of their own oocytes (Melnick et al., 2018). There can be numerous reasons; physiological or premature cessation of ovarian function, loss of ovarian function following chemotherapy or radiotherapy, poor oocyte quality, repeated failures of assisted reproductive technology (ART) using the patient’s own gametes, and genetic diseases which cannot be detected in the embryos by using preimplantation diagnosis strategies. This approach has progressively become ethically and legally accepted in the majority of countries. The procedure is based on the existence of at least three subjects. Usually, the three elements involved are the oocyte donor, the recipient (who receives the fertilised egg) and the recipient’s partner, although there is also the possibility of donated sperm. The donor undergoes controlled ovarian hyperstimulation in conventional IVF and, after oocyte retrieval, her oocytes (fresh or warmed after vitrification) are inseminated in vitro with the sperm of the recipient’s partner (or the male donor). The fertilised oocytes are then transferred to the hormonally synchronized endometrium of the recipient.

European statistics highlight that there is an increasing need for donated oocytes. Among 39 countries in Europe offering ART, a total of 56,516 egg donation cycles were reported in 2014, with a steep increase in treatment numbers since 2013 (+ 40.4%) (De Geyter et al., 2018).

As the number of potential recipients is continuously increasing, the major difficulty in establishing a donor oocyte program is the limited availability of donor subjects. Donor recruitment is challenging, since it depends on many factors . Donors usually need to be younger than 35 years old to reduce the risk of chromosomal abnormalities in oocytes (Munnè et al., 1995; Demko et al., 2016; La Marca et al., 2017). Moreover donors must not to have any contraindication to donate due to medical, genetic, or psychological reasons.

Many countries do not allow donors to be compensated financially, except for a reasonable reimbursement of their expenses and this will not make easy to promote the need of donating oocytes among the general female population. A possible solution to the lack of donors could be oocyte sharing, which means that women undergoing IVF for infertility could donate some oocytes to a recipient. Oocyte sharing, however, raises several ethical and medical concerns. A “shared” oocyte donation programme is limited by the very low number of oocytes available for donation and by the quality of the oocytes, which depends largely on donor’s age and, in the case of donation by an infertile woman, on her infertility etiology. Furthermore, IVF patients often wish to maximise their chances of pregnancy by using a significant number of their own oocytes and by cryopreserving supernumerary embryos for their own use (World Health Organization, 2002).

Unlike other countries with more restrictive laws, oocyte donation has been regulated in Spain since 1988. Spain is the world leader in organ donation and transplantation; this success is due to several factors, such as adequate legislation, widespread public campaigns, a systematic approach to potential donations in hospitals, the presence of a donation coordinator, and a social conscience that promotes organ donation, in which oocytes are included. The procedure is compensated with a flat-rate amount that takes into consideration several factors (i.e. anaesthesia risk, lost working days lost, treatment burden, possible complications). This amount, which is about 1,000 euros per donation (irrespectively of oocyte number), is the same in every clinic and has been suggested by the national bioethics committee. The donor’s identity is protected by anonymity and the greatest possible phenotype similarity between the donor and the recipient may be offered by an extensive donor bank of different nationalities which enables the matching of the phenotypical features of patients of different ethnicities.

While in Spain (and in Italy) the donor’s identity is protected by anonymity, in many other countries the debate about disclosure of donor conception is not new (Harper et al., 2016) While many appreciate that there may be many benefits associated to the use of known donors, there are concerns about the effect that withdrawal of anonymity may have on an egg donor recruitment programme.

## Low availability of donors may limit the accessibility to the treatment

When gathering and analysing data concerning ART regulations and policies in the European Union, it is easy to come to the conclusion that a lot of disparities (economic, ethnic, geographic, social and cultural) exist among countries and even within countries. These disparities often represent obstacles for accessing ART and can, indeed, contribute to inequalities between patients accessing reproductive health care services. Reproductive health could therefore be a fertile ground for so called “health tourism” or, in this specific case, “reproductive tourism.”. “Cross-border reproductive care” refers to patients travelling from their home country to another one in order to get fertility treatment through ART. The main drivers for fertility tourism are legal regulations, or lower price and higher success reported in foreign countries when compared to the home country. In countries in which donors’ availability is still insufficient to cover the therapeutic demands, patients could therefore be referred abroad for treatment.

Oocyte donation remains one of the most important unmet treatment needs in countries like Italy (Audibert and Glass, 2015). In Italy gamete donation has been forbidden from 2004 (Law 40/2004) to 2014, when the Italian Constitutional Court declared the unconstitutionality of the prohibition (decision n. 162/2014). Since then, more than 16,000 donor oocyte cycles have been performed (www.iss.it/pma, data from 2014 to 2017). Nevertheless, Italian patients and clinicians face the problem of very limited availability of oocyte donors at a local level. Every year thousands of couples travel abroad to seek treatments with heterologous gametes (Shenfield et al., 2010). It is well known that infertility and ART have an impact on the psycho-social well being of patients (Cousineau and Domar, 2007; Pasch et al., 2016), and the need to travel abroad to undergo reproductive care adds further emotional and practical complexity to the treatment, making the whole procedure even more burdensome (Culley et al., 2011; Hudson et al., 2011; Madero et al., 2017). Moreover, traveling abroad imposes additional financial strain associated with travelling expenses, while decreasing accessibility to the treatment.

## Shipment of vitrified oocytes or embryos: a solution to the problem?

Improvements in oocyte and embryo cryopreservation techniques have made it possible for reproductive physicians to respond to needs in new and unprecedented ways. Until a few years ago, the most used freezing technique, called slow freezing, provided disappointing results for oocyte cryopreservation (Bernard and Fuller, 1996) and had a low yield for embryo freezing, giving an average survival rate of around 52% (Debrock et al., 2015). The first birth following oocyte vitrification was reported in 1999 by an Australian-Italian team (Kuleshova et al., 1999), around ten years after the first pregnancies resulting from embryo vitrification (Gordts et al., 1990; Feichtinger et al., 1991). These techniques became widespread throughout the early 2000s, and represented a real breakthrough in reproductive biology and medicine. They in fact enabled oocyte and embryo cryopreservation to be achieved with excellent survival rates and pregnancy chances similar to those with fresh transfers (Cobo et al., 2008; 2010; Rienzi et al., 2010).

The practice of freezing oocytes and embryos is still increasing, and that can be clearly seen in the steep rise of the number of frozen embryo transfer (FET) treatments reported, which in 2014, for the first time, exceeded that of fresh transfers (De Geyter et al., 2018).

The revolution we are witnessing with the development of cryopreservation techniques is the freedom to move away from the standard sequence of ovarian stimulation-retrieval-transfer, to which we were previously bound, in order to provide the best chances of success (Massin, 2017). Oocyte vitrification has in fact radically changed donation programs, uncoupling the donation from the reception both from a spatial and a temporal point of view. This has led to the development of cryobanks of donated oocytes, with several recipient programmes completely based on the use of vitrified donor oocytes. Usually, donor oocyte cryobanks provide sets of a finite number of mature vitrified oocytes per recipient (usually six), claiming results similar to those obtained with the use of fresh oocytes (Parmegiani et al., 2019). Donor egg banking provides relative benefits in terms of treatment, such as scheduling flexibility, and can allow for better phenotypical matching between recipient and donor, especially in small programmes. Most importantly, donor oocyte banking has also made it possible to transfer oocytes across borders. This is a second revolution in the oocyte donation programme as it increases access to treatment, since women living in countries with low availability of donors can use oocytes donated abroad.

However, while some studies report comparable success rate when using vitrified and fresh oocytes (Cobo et al., 2015), this does not seem to be confirmed in larger national cohorts (Kushnir et al., 2018). In a recent retrospective analysis, including 30,160 IVF cycles with either fresh or cryopreserved donor oocytes from 2013 to 2015, fresh donor oocytes have been indicated to provide significantly higher live birth rate (LBR) per recipient cycle than cryopreserved donor oocytes (51.1 versus 39.7%); fresh oocyte donation must, therefore, still be considered the gold standard in oocyte donation according to the authors (Kushnir et al., 2018). Experience of different operators and centres in freezing and warming oocytes may largely impact on the survival rate of oocytes, hence explaining the huge variability in live birth rates reported by different centres.

Compared to oocytes, the performance of embryo cryopreservation seems to be more reliable as it shows reduced centre-dependence since results published in the literature are very consistent (Chen et al., 2016; Wong et al., 2017; Shi et al., 2018; Zhang et al., 2018a; Zhang et al., 2018b). Because of the very high survival rate after thawing and the high implantation rate of vitrified embryos, the proportion of frozen embryo transfer (FET) cycles was estimated to contribute 24.7% of the total number of transfers performed in Europe in 2014 (De Geyter et al., 2018). The LBR from elective FET has been reported to be comparable to fresh ET (50.2 and 48.7%, respectively) (Shi et al., 2018).

**Figure 1:**
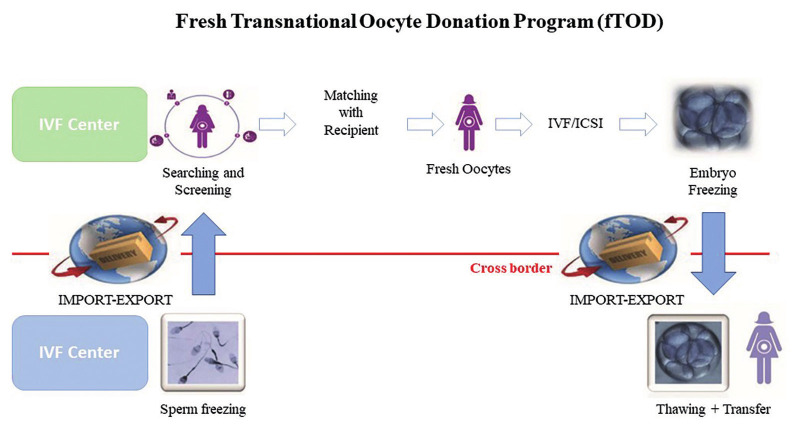
— A schematic representation of the fresh transnational oocyte donation programme (fTOD).

Recently, a novel approach based on frozen embryos instead of frozen oocytes to satisfy the increasing request of crossborder oocyte donation has been proposed by our group (La Marca et al., 2019a) (Figure 1).

**Figure 2:**
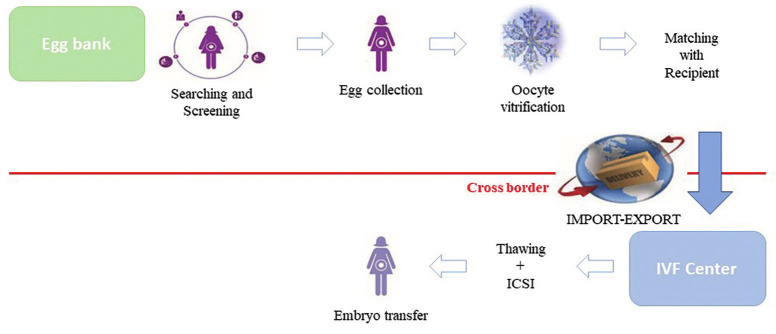
— A schematic representation of the vitrified transnational oocyte donation programme (vTOD).

Unlike the routine of most cryobanks (Figure 2),it allows for the fertilisation of fresh instead of vitrified oocytes. With this approach, the authors seek to reduce travelling costs for the patients, as well as avoid further emotional and practical complexity to the treatment.

In our recent article based on 630 patients undergoing oocyte donation cycles with frozen embryos from fresh oocytes, the live birth delivery rate was 30.6% after the first embryo transfer. The conservative and optimistic live birth delivery rate after the second frozen embryo transfer were 39.3% and 48.7%, respectively. Importantly, after the first and the second embryo transfer, the percentage of patients who still had at least one cryopreserved embryo was 85.3% and 73.1%, respectively (La Marca et al., 2019a).

Recently, another Italian experience but this time based on the shipment of vitrified eggs has been published (Rienzi et al., 2020). In this study including 273 patients, a mean of 7.3 oocytes per patient were warmed and the survival rate was 86%. Hence a mean of 6.3 oocytes were available for patients, with 16% of them having ≤4 oocytes to be inseminated. The live birth delivery rate after the first embryo transfer was 28.5%, hence very similar to what reported by our group (30.6%) when using fresh instead of frozen oocytes. When calculating variables affecting the cumulative live birth delivery rate by multivariate logistic analysis, authors found that the number of warming and survived oocytes was a strong and significant predictor (Rienzi et al., 2020). Most importantly, the authors have calculated the oocyte-to-baby rate: in their study a total 1982 oocytes were warmed and 125 babies were born, hence the oocyte-to-baby rate was 6.3% with an estimated number of 15.8 oocytes required per live birth. In our study a total of 4292 oocytes lead to the live birth of 307 babies, hence the oocyte-to- baby rate was 7.1%, and an estimated number of 14 oocytes was required per live birth.

The transnational fresh oocyte donation (TOD) programme seems to provide several advantages over the shipment of vitrified oocytes. We think that the use of frozen embryos from fresh oocytes could be associated with a higher cumulative LBR per started cycle when compared to a similar number of vitrified oocytes; this may be due to true loss of developmental ability of vitrified oocytes, imperfect warming techniques in the receiving centres and the difficulty of shipping a tailored number of assigned oocytes to the patient’s needs. In the last reports of the Italian IVF registry, data relative to the pregnancy rate with the use of vitrified donor oocytes have been reported. In 2017, 3089 such cycles were performed (more than twice the cycles performed in 2015) with a pregnancy rate per couple treated of 36.0% (www.iss.it/pma) (Table I). The strategy based on the use of fresh oocytes and transfer of cryopreserved embryos, on the other hand, has proven to be consistent and efficient (the CPR and LBR per couple treated were 40.3 and 30.7%, respectively, considerably higher than those reported with the use of vitrified oocytes). Of note is that the rate of cycle cancellation has been of 3.8 % in the TOD program while it was higher (7.3%) when frozen oocytes were used.

**Table I:** — Data from Italian IVF registry on female heterologous gamete donation (2015 – 2017).

Donor fresh oocytes				Donor frozen oocytes			Frozen embryos from gamete donation		
2015		2016	2017	2015	2016	2017	2015	2016	2017
Centers performing at least one donation cycle, N	19	9	9	54	68	68	33	53	72
Couples treated, N	107	143	60	1113	2531	2828	369	1434	2266
Cycles initiated, N	110	143	60	1198	2758	3089	420	1735	2783
Transfers, N	100	137	45	1106	2513	2863	409	1709	2677
Clinical pregnancies obtained, N	40	49	15	341	833	1018	132	551	914
Live births, N	/	/	/	/	/	740	/	/	695
Pregnancy rate per cycle initiated, %	36.4	34.3	25.0	28.5	30.2	33.0	31.4	31.8	32.8
Pregnancy rate per transfer, %	40.0	35.8	33.3	30.8	33.1	35.6	32.3	32.2	34.1
Live birth rate per cycle initiated, %	/	/	/	/	/	24.0	/	/	25.0
Live birth rate per transfer, %	/	/	/	/	/	25.8	/	/	26.0
Pregnancy rate per couples treated, %	37.4	34.3	25.0	30.6	32.9	36.0	35.8	38.4	40.3
Live birth rate per couple streated, %	/	/	/	/	/	26.2	/	/	30.7
Cancellation rate, %	/	/	/	/	/	7.3	/	/	3.8

/ = data not available.

A recent updated analysis of our clinical activity including more than eight hundred patients showed a live birth rate per transfer as high as 37.2% after the first embryo transfer (Table II). Of note is that the vast majority of patients still have some cryopreserved embryos remaining, hence the cumulative live birth rate is expected to be even higher that what reported.

**Table II:** — Outcome of 896 patients at the first embryo transfer at the first egg donor cycle according to the fTOD programme.

TOD cycles	N =896
Embryo frozen	3467
Embryos warmed	1448
Embryos survived	1428
Surivival rate	98.6%
Beta+ rate	51.5%
CPR rate	43.2%
Live Birth rate	37.2%
Delivery rate	33.1%
Single Live birth at delivery	81%
Twins live birth at delivery	19%

The use of fresh oocytes enables personalised care for the oocyte recipient, with a flexible number of oocytes assigned. This personalisation is more complicated than the procedure carried out with fixed pre-prepared packs of oocytes, and this is probably the reason why cryopreserved oocyte donation cycles may perform more poorly than fresh donor oocytes. When using frozen oocytes, in fact, a standard number of 6-8 oocytes is usually assigned to a couple. In our approach, on the contrary, clinicians have the possibility to personalise the number of oocytes, for example if a male factor or low fertilisation risk is assessed. In our already published experience (La Marca et al., 2019a), we reported that the number of fresh oocytes assigned to our patients ranged from 6 to as high as 11. In the end, this might lead to a total higher availability of embryos for the patients.

In any case, shipment of both gametes and embryos has ultimately very positive aspects from a patient’s perspective, as it leads to easily accessible treatment in the patients’ own cultural and medical context, and to the reduction of expenses for traveling abroad.

There have been a few criticisms towards this approach. First, it has been suggested that the use of frozen embryos is associated with decreased pregnancy rates compared to the one reported for oocyte donation cycles (when based on fresh embryo transfer) in the principal national and international IVF registries (Requena et al., 2019). Furthermore, potential setbacks linked to the transport of the frozen samples, with potential loss of 100% of the eggs/embryos in transit should be taken into account. It has also been said that since the utilisation of fresh material rather than frozen could be associated with higher pregnancy rates, international travel to perform fresh transfer should be preferred. We argue that it is methodologically very difficult to compare the results from different registries (La Marca et al., 2019b). The issue of a possible loss of the frozen embryos while they are en route is significant, since it could carry profound personal, ethical and legal consequences, but the convenience of storing frozen embryos remains and must remain unaffected by accidents. Parmegiani et al. (2019) have also suggested that the suboptimal results reported for the year 2015 in gamete donation cycles were to be expected (Table I), since 2015 was the first full year of gamete donation re-legalisation in Italy. In their experience, the clinical research group reported 102 embryo transfer procedures in 100 patients (with 164 blastocysts transferred) during the same period as our investigation (January 2016 - February 2018). They imported 8 frozen eggs per patient and the mean number (±SD) of oocytes injected was in the end comparable to that of our study (6.4±1.5 vs 6.8±1.0). Beyond the small sample size the data reported shows that while 8 vitrified oocytes are imported per patient, the average number of injected oocytes per cycle in the authors’ clinic was 6.4, indicating a survival rate of approximately 80%, well below what reported by using frozen embryos (98.5% in our own study). Since donor oocytes are a limited resource, and there is a responsibility for the IVF provider to ensure access to treatment, the live birth per oocyte used is a parameter of interest for IVF providers. Given the above reported figures, the fresh TOD becomes a more efficient strategy, eliminating the loss of oocytes in the process of vitrification/warming. It is well known that oocyte freezing may be associated to a non-negligible possible impairment of oocyte quality after thawing, and even if vitrification is considered not to impact on oocyte quality and implantation rate of the resulting embryo, this technique is more complex and linked to variability in results (Seshadri et al., 2018; Youm et al., 2018). Finally, as proposed by others (Kushnir et al., 2018), it cannot be excluded that oocyte donor selection by commercial donor oocyte banks may not be as rigid as donor selection for fresh donor cycles by infertility centres and this may have some consequences in terms of success rates.

According to our national data collected from 2015 to 2017, there has been a widespread take-up of TOD, indirectly confirming its efficacy: in this period almost 5,000 and more than 7,000 cycles using frozen embryos from fresh oocytes and vitrified donor oocytes respectively were initiated (Table I). It is important to notice that of the 2783 cycles using frozen embryos from fresh oocytes in 2017, 73.8% have been done in large clinics (with more than 500 IVF/year). Conversely, of 3,089 cycles using imported vitrified oocytes, less than 50% were done in centres with > 500 cycles, indicating a role for experience and organization of the IVF centre in choosing the strategy for the egg donation cycle (La Marca et al., 2019b).

## Conclusions

Transportation of eggs and embryos between different international laboratories has permitted an increased accessibility to oocyte donation in countries with low availability of donors. In our experience, many couples have declared that they would have never travelled abroad for an ART treatment due to high costs of flights and hotels, insufficient knowledge of foreign languages, and low experience of travelling abroad. For these couples, the success rate of 40-50% with TOD must be compared to 0%, which is resulting from the non-adherence to the egg donation programme (La Marca et al., 2019b). From this point of view the most relevant clinical advantage of the TOD programme has been the possibility of bringing oocyte donation to infertile couples.

Of course, TOD may be based on fresh or frozen eggs; so we propose to define “fresh transnational oocyte donation” (fTOD), the programme based on shipment of sperm and retrieval of frozen embryos, and “vitrified transnational oocyte donation” (vTOD), the one based on importation of vitrified eggs (Figure 1 and Figure 2).We can hypothesise that, according to data published to date, the number of eggs needed to obtain an embryo is slightly higher when vitrified oocytes are used, and probably a higher number of embryos resulting from the use of a fixed number of fresh versus vitrified eggs may lead to a higher cumulative LBR. Of course, this is at this moment our hypothesis and needs to be demonstrated by upcoming studies on this subject.
